# Bee larvae ameliorate andropause-like symptoms via a hormone-independent, antioxidant mechanism

**DOI:** 10.3389/fphys.2026.1850114

**Published:** 2026-05-29

**Authors:** Takashi Ito, Nobuaki Okumura, Takumi Oti, Hirotaka Sakamoto

**Affiliations:** 1 Department of Biology, Faculty of Environmental, Life, Natural Science and Technology, Okayama University, Okayama, Japan; 2 Institute for Bee Products & Health Science, Yamada Bee Company, Inc., Okayama, Japan

**Keywords:** anxiety, bee larvae, late-onset hypogonadism, oxidative stress, sexual behavior

## Abstract

Late-onset hypogonadism (LOH), also known as the male menopause, is characterized by a decline in sexual function as well as various physical and psychological symptoms, including anxiety. Although bee larvae have historically been utilized as a traditional food and medicine, their efficacy and physiological mechanisms of action against male menopausal symptoms remain unclear. In this study, we investigated the effects of bee larvae (BL) on sexual and anxiety-like behaviors using two rodent models of the male menopause: aged rats and castrated mice. In the aged rat model (64 weeks old), dietary BL supplementation for 4 weeks significantly attenuated the age-associated decline in ejaculation frequency compared to controls, while no significant effects were observed on mount or intromission frequencies. Notably, plasma analysis revealed no significant differences in testosterone or dihydrotestosterone levels between the BL and control groups. To elucidate the underlying mechanism, we evaluated sexual function using a castrated mouse model. While BL supplementation did not affect sexual behavior in intact mice, post-castration BL treatment significantly shortened intromission latency without altering mount frequency. In the elevated plus maze test, BL significantly alleviated castration-induced anxiety-like behaviors and improved exploratory activity. Furthermore, *in vitro* assays demonstrated that the BL extract exerts potent protective effects against oxidative stress, a pathological factor contributing to both erectile dysfunction and anxiety. These results suggest that BL improves erectile function and anxiety via hormone-independent mechanisms, potentially by mitigating oxidative stress in vascular and neural tissues. Thus, bee larvae represent a promising functional food for ameliorating the multi-faceted physical and psychological symptoms associated with male menopause.

## 1 Introduction

In a rapidly aging society, maintaining the Quality of Life (QOL) stands as a priority in modern healthcare. Within this demographic, late-onset hypogonadism (LOH) has emerged as a critical health challenge (Corona et al., 2013). Characterized by an age-related decline in circulating androgens, LOH manifests through a broad spectrum of andropause-like symptoms, ranging from diminished sexual function to physical lethargy, sleep disturbances, and psychological instability, including anxiety and depression. Notably, while sexual desire often persists in middle-aged and elderly men, the physiological decline in sexual function can severely compromise self-esteem and QOL, driving a significant number of individuals to seek medical intervention (Corona et al., 2013).

Key factors contributing to the decline in male QOL include erectile dysfunction (ED) and psycho-emotional instability, particularly anxiety. Epidemiological studies indicate that the prevalence of ED increases with age, affecting approximately 40% of men in their 40s and 50% of men in their 50s (Ferrini et al., 2017). Systemic oxidative stress has been identified as a significant contributor to this age-related increase in ED (Agarwal et al., 2006). Oxidative stress plays a pivotal role in the pathogenesis of atherosclerosis, diabetes, hypertension, and hypercholesterolemia, leading to vascular endothelial dysfunction (Agarwal et al., 2006). Notably, penile arteries, having a significantly smaller diameter than coronary or internal carotid arteries, are particularly susceptible to oxidative stress-induced endothelial damage. The resulting reduction in cavernosal blood flow is considered a primary cause of ED (Ferrini et al., 2017; Kaltsas et al., 2024). Furthermore, elevated oxidative stress is intricately linked to the pathophysiology of psychiatric symptoms, including anxiety and depression (Fedoce et al., 2018; Correia et al., 2023). Given their high oxygen consumption, cerebral neurons are highly vulnerable to oxidative stress; oxidative damage can disrupt neurotransmitter homeostasis, potentially leading to emotional dysregulation (Fedoce et al., 2018; Correia et al., 2023). Therefore, the augmentation of oxidative stress associated with aging and androgen deficiency may serve as a common etiological factor simultaneously driving two distinct symptoms: ED and anxiety.

Bee larvae (BL), comprising both the larvae and pupae of bees, have historically been consumed worldwide as a highly nutritious food source (Guiné et al., 2022). They possess an exceptionally high nutritional value, containing a diverse array of nutrients including proteins, carbohydrates, and lipids, as well as vitamins, minerals, essential amino acids, and unsaturated fatty acids (Finke, 2005). In recent years, beyond their rich nutritional composition, studies have reported their physiological benefits, such as antioxidant and immunomodulatory effects (Moraru et al., 2024; Li et al., 2020). Furthermore, clinical studies have suggested their efficacy in improving hearing and ameliorating symptoms associated with male menopause (Aoki et al., 2012; Keishi et al., 2020). Specifically, in an open-label, single-arm clinical trial targeting male menopausal symptoms in 11 men, supplementation with BL for 12 weeks has been observed to improve scores on the Aging Males’ Symptoms (AMS) scale and the Sexual Health Inventory for Men (SHIM), while also reducing fatigue and depressive symptoms. However, the precise mechanisms underlying the functional properties of BL and their therapeutic effects on male menopausal symptoms and erectile function remain to be fully elucidated. Therefore, the present study focused on BL as a potential therapeutic agent for improving male sexual function. This study aimed to verify the mechanism of action of BL, specifically focusing on the suppression of oxidative stress, using a castrated mouse model of male menopause. This model reproduces the decline in erectile function and anxiety-like symptoms induced by androgen deficiency.

## 2 Material and methods

### 2.1 Animals and ethics

All procedures involving animals were conducted in accordance with the principles outlined in the ARRIVE guidelines. All efforts were made to minimize animal suffering and the number of animals used for the studies. Although a formal *a priori* power analysis was not conducted, the sample sizes for the rat and mouse experiments (*n* = 12 and *n* = 10 per group, respectively) were determined based on established protocols from previous comparable behavioral studies. This approach balanced sufficient statistical power to detect meaningful behavioral changes with strict adherence to the 3Rs principle, minimizing animal use.

Rat experiment: The study protocol for the rat experiment was reviewed and approved by the Institutional Animal Care and Use Committee of the Animal Reproduction Institute (Approval No.: Sho-3-33) and was conducted at the same institute (Kasumigaura, Ibaraki, Japan). Wistar-Imamichi rats were procured from the Animal Reproduction Institute.

Mouse experiment: The study protocol for the mouse experiment was approved by the Animal Care and Use Committee of Okayama University (Approval No.: OKU-2023607). This experiment was conducted at Okayama University (Okayama, Japan). ICR male and female mice were purchased from Japan SLC Inc. (Shizuoka, Japan).

### 2.2 Preparation of bee larvae powder and diets

Enzyme-digested bee larvae (BL) powder, prepared from 19–20-day-old larvae (*Apis mellifera*) treated with protease, was supplied by Yamada Bee Company, Inc. (Okayama, Japan). The experimental diets were prepared by mixing the BL powder into a commercial basal diet, MF (Oriental Yeast Co., Ltd., Tokyo, Japan), at a concentration of 6.4% (w/w). As no prior animal studies using this specific BL powder were available, the dose was determined by extrapolating the effective dose from a previous human clinical trial (Keishi et al., 2020) using body surface area (BSA) normalization, adjusted for the higher metabolic rate of rodents to ensure sufficient exposure.

### 2.3 Rat experiment (aging model)

#### 2.3.1 Experimental design and housing conditions

A total of 24 Wistar-Imamichi male rats (*n* = 12 per group; 64 weeks old at the start of feeding) and female rats (9 weeks old) were procured from the Animal Reproduction Institute and used for this study. All animals were housed under controlled temperature (23 ± 3°C) and maintained on a 12 h light/dark cycle with *ad libitum* access to food and water. Male rats were kept in individual cages. To ensure consistent sexual performance throughout the experiment, male rats were sexually experienced before the feeding period. Healthy male rats were divided into two groups based on equal pre-feeding copulatory performance: the Control group (MF diet) and the BL group (BL diet). The feeding period lasted 4 weeks.

#### 2.3.2 Sexual behavior test

It is well-established that sexual behavior declines with advancing age in male rats (Smith et al., 1992; Hokao et al., 1992). Sexual behavior was observed before and after the 4-week feeding period. For testing, sexually experienced male rats were paired 1:1 with sexually receptive, naïve female rats. Females were confirmed to be in behavioral estrus *via* the manual lordosis reflex on the day of testing. Observations were conducted during the dark cycle. Sexual behavior was recorded for 60 min and quantified using the following established Heimer indices: number of mounts, number of intromissions, number of ejaculations, mount latency, intromission latency, and ejaculation latency (Heimer and Larsson, 1967).

#### 2.3.3 Hormone measurement

Following the final sexual behavior test, all male rats were fasted for 16 h. Blood samples were collected under isoflurane anesthesia from the caudal vena cava. Plasma was obtained and stored at −80°C until hormonal analysis. Plasma concentrations of testosterone (T) and dihydrotestosterone (DHT) were quantified by ASKA Pharmaceutical Co., Ltd. (Tokyo, Japan) using a highly sensitive liquid chromatography–tandem mass spectrometry (LC-MS/MS) method. According to the manufacturer’s validation data, the limit of quantification (LOQ) for both T and DHT was 5 pg/mL. The intra- and inter-assay coefficients of variation (CVs) were 1.4–2.8% and 3.4–5.1% for T, and 4.4–9.6% and 6.9–12.2% for DHT, respectively.

### 2.4 Mice experiment (castration model)

#### 2.4.1 Experimental design and housing conditions

A total of 20 male (n = 10 per group) and 20 female ICR mice (8–10 weeks old) were purchased from Japan SLC Inc. (Shizuoka, Japan). Mice were housed separately by sex (3–6 per cage) under controlled temperature (23 ± 3°C) and maintained on a 12 h light/dark cycle with *ad libitum* access to food and water. To determine food intake in this group-housed setting, a pre-weighed amount of food was provided to each cage. The remaining food was weighed, and the total consumed amount was divided by the number of mice per cage to calculate the mean daily food intake per individual. To ensure consistent sexual motivation and performance, male mice were sexually pre-conditioned before the observation period; this involved providing them with at least two sexual experiences, including one ejaculation per week. This procedure was conducted to prevent the level of prior sexual experience from confounding the experimental results. Male mice underwent castration under isoflurane anesthesia upon completion of the 12-week sexual behavior evaluation period. Following castration, the mice continued to receive their assigned BL or control diets for an additional 4 weeks (until week 16), and the elevated plus maze test was performed at the study endpoint (week 16).

#### 2.4.2 Sexual behavior test

Sexual behavior tests were performed before and after castration, following the general protocol for parameter enumeration described for the rat experiment (Section 2.3.2). To eliminate variability from the natural estrous cycle and bring stimulus females into behavioral estrus, female ICR mice were ovariectomized (OVX). This standard procedure in sexual behavior research ensures that observed changes in copulatory behavior are attributable solely to male performance. Estradiol benzoate (EB, 25 µg/0.05 mL sesame oil) was subcutaneously injected 48 h prior to testing, followed by progesterone (P, 250 µg/0.05 mL sesame oil) subcutaneously injected 3–6 h before testing. Sexual behavior was recorded for 60 min. The parameters evaluated were the number of mounts, the number of intromissions, mount latency, and intromission latency.

#### 2.4.3 Elevated plus maze test

To evaluate anxiety-like and exploratory behaviors, the elevated plus maze (EPM) test was conducted at week 16 of the experiment. The test apparatus consisted of a plus-shaped maze elevated 75 cm above the floor, with two open arms and two closed arms (50 cm × 10 cm). The closed arms were enclosed by 40 cm high walls. Mice from each group were randomly selected and placed at the center of the maze, facing an open arm. Their behavior was recorded on video for 5 min. After each test, the apparatus was thoroughly wiped with 70% ethanol to eliminate any residual olfactory cues. The video recordings were then manually scored to quantify the following parameters: time spent in the open arms, total number of arm entries (open and closed arms), and the number of head-dips from the open arms. Time spent in the open arms and head-dips are considered indicators of anxiety-like and exploratory behaviors, respectively, while the total number of arm entries serves as an index of general locomotor activity.

### 2.5 *In vitro* oxidative stress protection assay

Human neonatal dermal fibroblasts (NB1RGB) were obtained from RIKEN BRC through the National Bioresource Project of the MEXT, Japan. Cells were maintained in MEM α (Thermo Fisher Scientific, Waltham, MA, USA) supplemented with 10% fetal bovine serum (FBS) and used within three passages. To prepare the test sample, BL powder was suspended in water and extracted by sonication for 30 min. For the viability assay, cells were seeded into 96-well plates at a density of 1.0×10^4^ cells per well. After 24 h of incubation, the medium was replaced with MEM α containing 0.1% FBS and the BL extract. One hour later, oxidative stress was induced by adding hydrogen peroxide (H_2_O_2_; Fujifilm Wako Pure Chemical Corporation, Osaka, Japan) to a final concentration of 200 µM. The cells were cultured for an additional 1 h, washed with PBS, and incubated in normal growth medium for 24 h. Cell viability was assessed using CellTiter-Glo 2.0 (Promega, Madison, WI, USA) according to the manufacturer’s instructions. Luminescence was measured using a microplate reader (PerkinElmer, Waltham, MA, USA). The experiments were performed in five independent trials (*n* = 5). In each trial, samples were assayed in triplicate wells, and the average value was calculated and used as a single data point.

### 2.6 Statistical analysis

All data are presented as the mean ± standard error of the mean (SEM). Statistical analyses were performed using GraphPad Prism version 10.6.0 (GraphPad Software, San Diego, CA, USA), and behavioral analyses were conducted in a blinded manner where applicable. Paired *t*-tests were used to evaluate within-group changes (pre- vs. post-feeding for rats; pre- vs. post-castration for mice). Unpaired *t*-tests were used for between-group comparisons (Control vs. BL) at the study endpoints, including sexual behavior parameters (week 4 for rats; week 16 for mice), EPM indices, and hormone levels. For the *in vitro* assays, multiple group comparisons against the H_2_O_2_-alone group were performed using one-way analysis of variance (ANOVA) followed by Dunnett’s *post hoc* test. A *P*-value < 0.05 was considered statistically significant.

## 3 Results

### 3.1 Effects of BL administration on sexual behavior in aged rats

**Figure 1:**
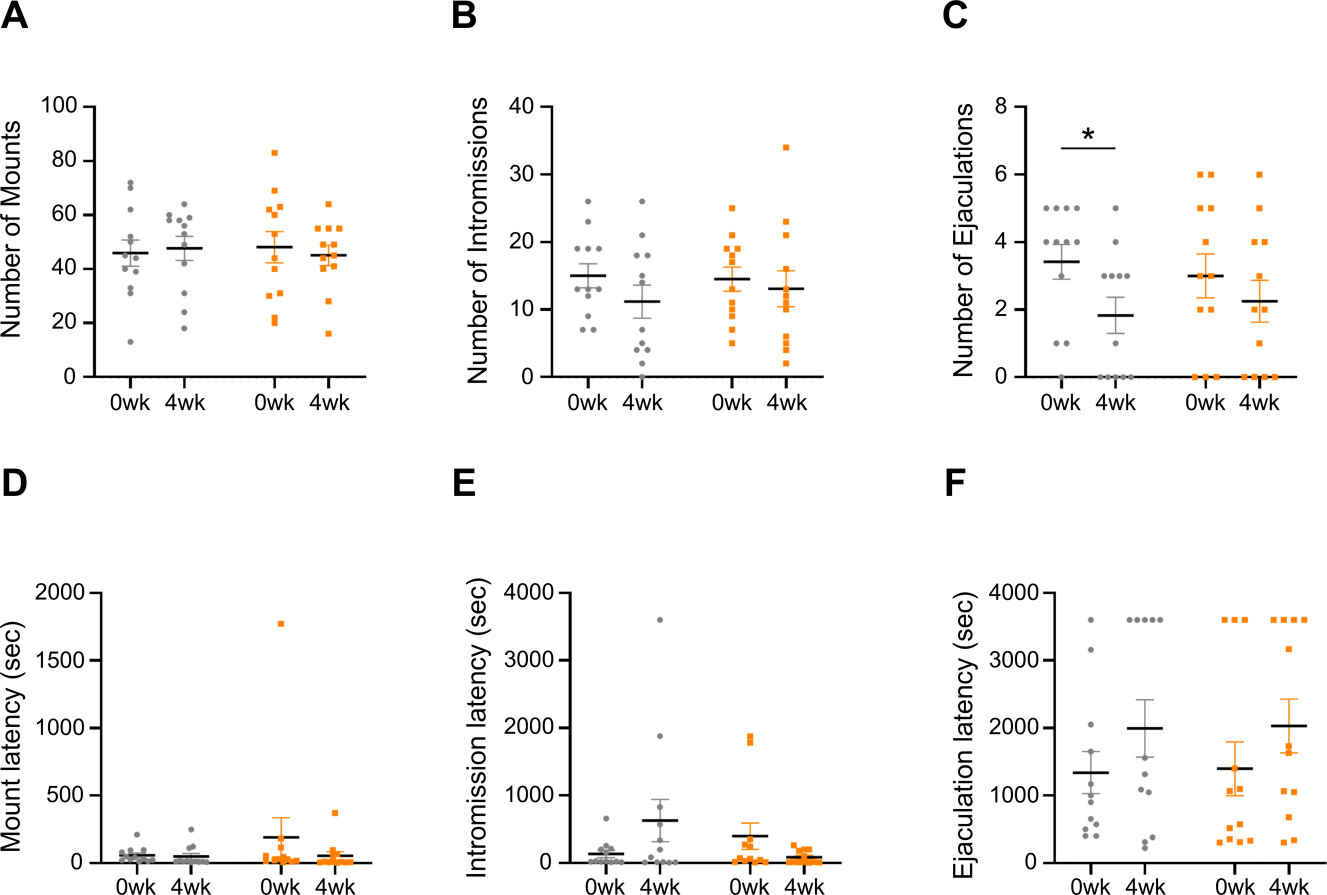
Effects of administration of extracts of bee larvae on sexual behavior parameters in aging rats. Male Wistar-Imamichi rats (64 weeks old) were administered the Control (gray) or BL (orange) diet for 4 weeks. Sexual behavior was evaluated before (0 weeks) and after the feeding period. **(A)** Mount frequency, **(B)** Intromission frequency, **(C)** Ejaculation frequency, **(D)** Mount latency, **(E)** Intromission latency, and **(F)** Ejaculation latency are shown. Data are presented as the mean ± standard error of the mean (SEM). Statistical significance was determined using two-way analysis of variance (ANOVA) followed by Bonferroni *post hoc* correction. *P < 0.05.

**Figure 2:**
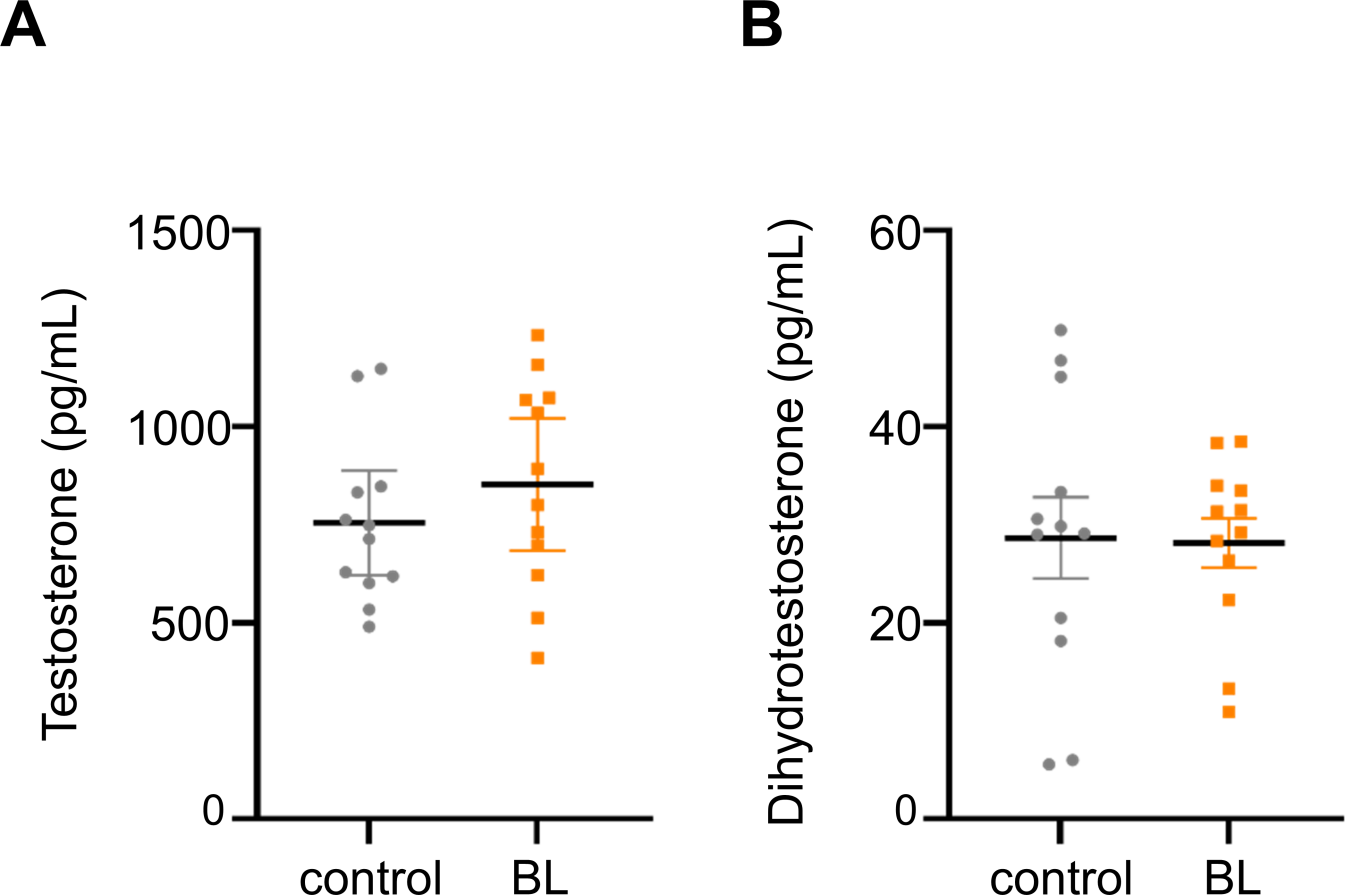
Plasma androgen levels after four weeks of bee larvae extract administration in aging rats. Plasma concentrations of **(A)** Testosterone (T) and **(B)** Dihydrotestosterone (DHT) were quantified in aging male rats after 4 weeks of feeding the Control (gray) or BL (orange) diet. The hormones were quantified using the LC-MS/MS method. No significant differences were observed between the Control and BL groups for either T or DHT levels. Data are presented as the mean ± standard error of the mean (SEM). Statistical significance was determined using Welch’s t-test.

The effects of dietary BL supplementation for four weeks on sexual behavior in aged rats were evaluated. Comparing sexual parameters before and after the four-week period, no significant changes were observed in the frequency or latency of mounts and intromissions in the Control group (**Figure 1**). Although ejaculation latency numerically increased in both groups, the changes did not reach statistical significance (Control: *P* = 0.0687, BL: *P* = 0.0796, **Figure 1F**). Notably, while a significant decline in ejaculation frequency was observed in the Control group (*P* = 0.0067, **Figure 1C**), no such change was detected in the BL group, suggesting the preservation of this function. Furthermore, quantification of plasma androgen levels after four weeks revealed no significant differences in testosterone or dihydrotestosterone concentrations between the Control and BL groups (**Figure 2**). These findings suggest that BL administration may help suppress the age-associated decline in sexual behavior in rats without affecting androgen levels.

### 3.2 Effects of BL administration on sexual behavior in castrated mice

Following the findings in the aged rat model suggesting that BL may attenuate the decline in sexual behavior, these effects were further verified using a castrated mouse model, which reproduces a state of androgen deficiency. This model is well established for studying symptoms resembling human male menopause, as the depletion of testosterone leads to a time-dependent decline in sexual behavior. In the present study, to validate this model and assess the efficacy of BL, sexually experienced male mice were administered a BL diet for 12 weeks. Subsequently, castration was performed, and the effects were investigated for an additional 4 weeks post-surgery.

**Figure 3:**
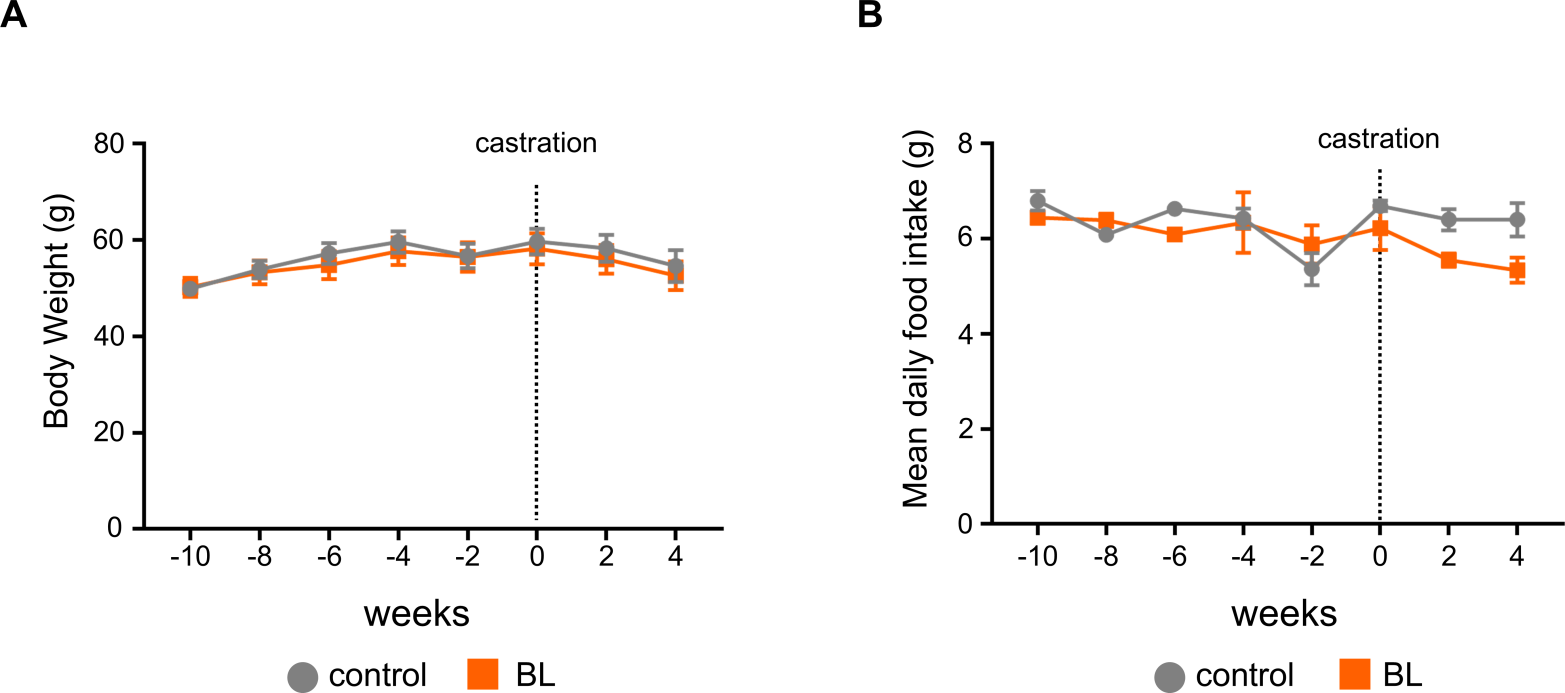
Effects of bee larvae extract administration on body weight and food intake in mice. Male mice were fed either a control diet or a BL diet for 12 weeks. The control group is represented by gray bars, while the BL-administered group is represented by orange bars. The arrow indicates the time of castration. **(A)** Animal body weight and **(B)** Food intake are shown. Data are presented as the mean ± standard error of the mean (SEM). Statistical significance was determined using two-way analysis of variance (ANOVA).

**Figure 4:**
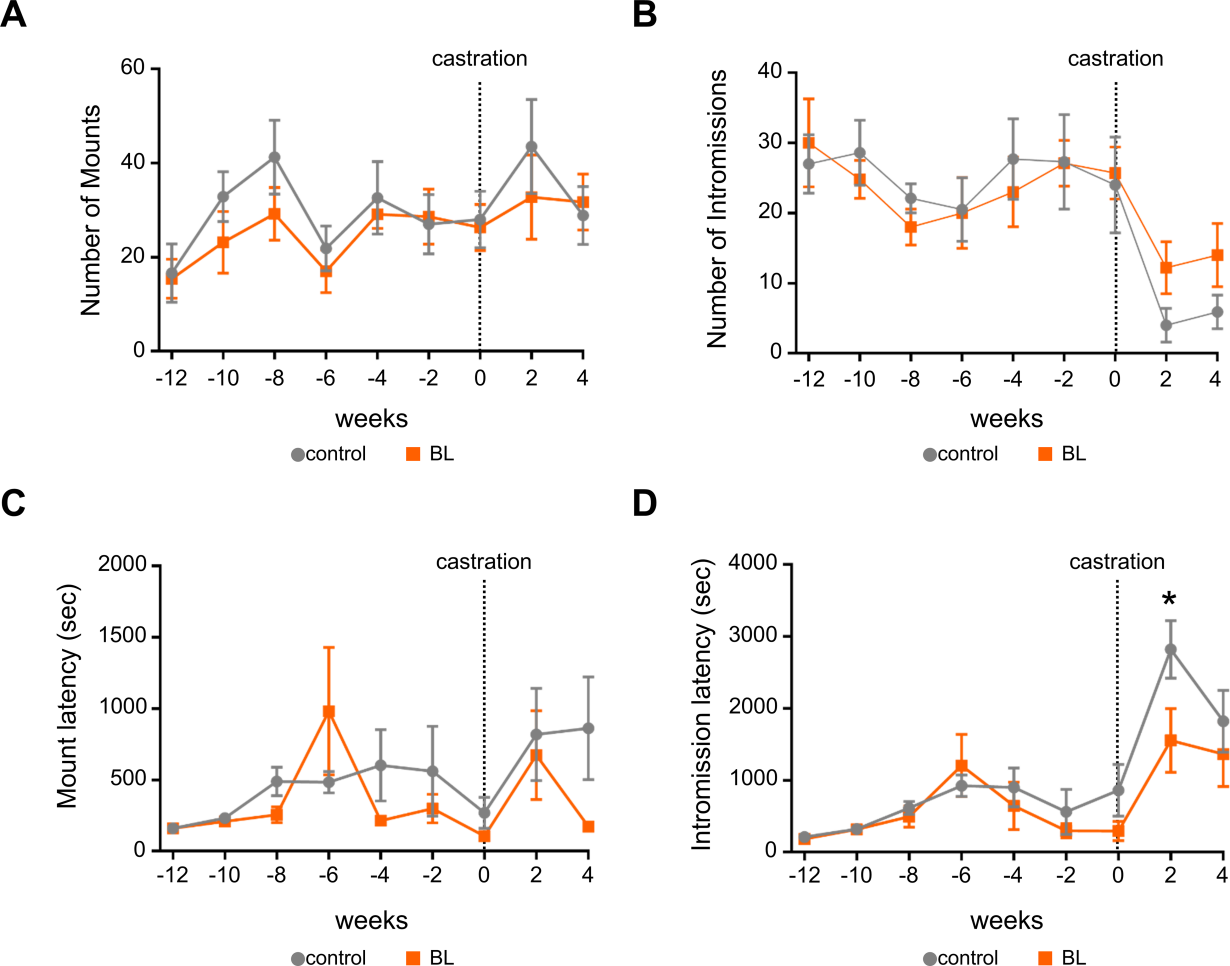
Effects of bee larvae extract administration on sexual behavior in intact and castrated mice. Male mice were fed either a control diet or a BL diet for 12 weeks. Sexual behavior was evaluated before castration (weeks 0–12). Subsequently, mice were castrated, and sexual behavior was evaluated for an additional 4 weeks (weeks 12–16). The control group is represented by gray bars, while the BL-administered group is represented by orange bars. **(A)** Mount frequency, **(B)** Intromission frequency, **(C)** Mount latency, and **(D)** Intromission latency are shown. Data are presented as the mean ± standard error of the mean (SEM). Statistical significance was determined using two-way analysis of variance (ANOVA) followed by Bonferroni *post hoc* correction. *P < 0.05.

Over the 12-week pre-castration period, no significant differences were observed in body weight or food intake between the Control and BL groups (**Figure 3**). Similarly, at the evaluation point 4 weeks post-castration, no significant differences were detected in these parameters. Subsequently, the effects of BL administration on sexual behavior were investigated using the frequency and latency of mounts and intromissions as key indicators. Prior to castration, no significant differences were observed between the Control and BL groups in either the frequency or latency of mounts and intromissions. However, regarding the castration-induced decline in sexual behavior, the BL group exhibited a significant shortening of intromission latency (*P* = 0.016) compared to the Control group at 2 weeks post-castration (week 14) (**Figure 4**). No significant differences were observed in the other parameters, specifically mount frequency, mount latency, and intromission frequency.

### 3.3 Effects on anxiety-like symptoms associated with male menopause

**Figure 5:**
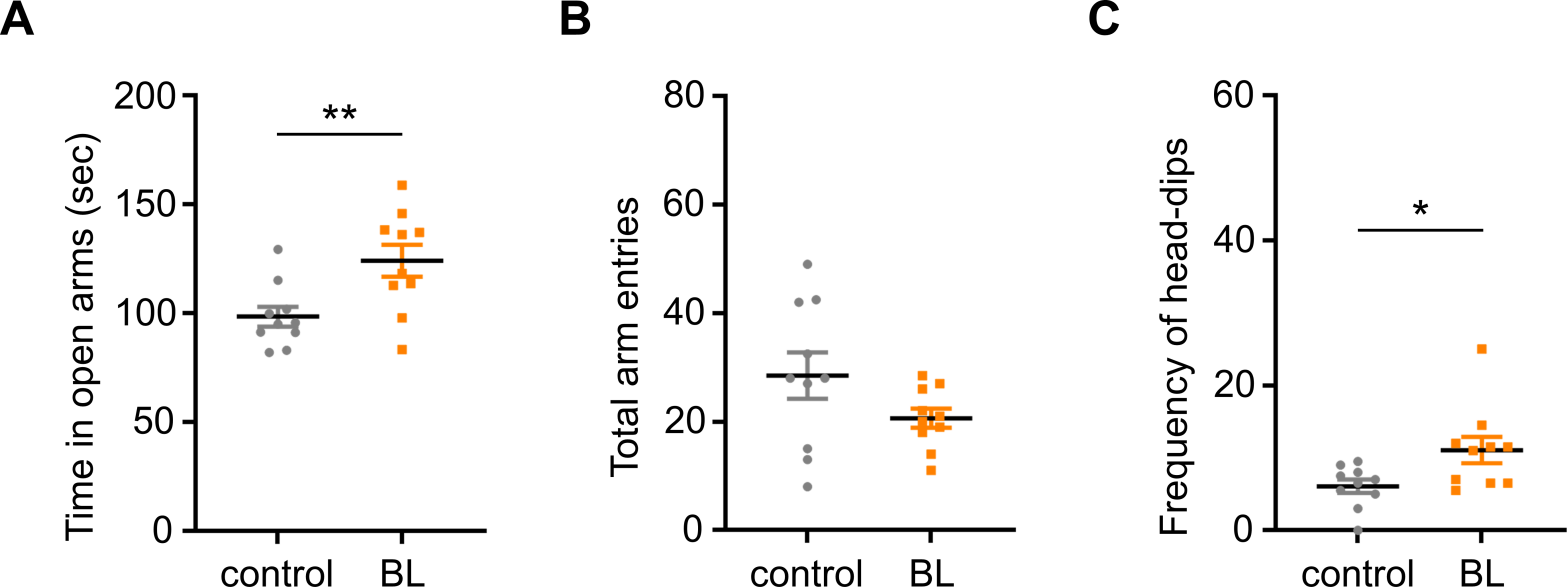
Effects of bee larvae extract administration on anxiety-like and exploratory behaviors in castrated mice. Mice were subjected to the elevated plus maze test at week 16 of the experiment. The figure shows **(A)** the total time spent in the open arms, **(B)** the total number of arm entries, and **(C)** the number of head-dips from the open arms. Data are presented as the mean ± standard error of the mean (SEM). Statistical analysis was performed using an unpaired *t*-test. *P < 0.05, **P < 0.01.

The elevated plus maze (EPM) test was conducted at 4 weeks post-castration, a time point corresponding to the established androgen depletion, to investigate the effects of BL administration on anxiety-like and exploratory behaviors (**Figure 5**). Compared to the Control group, the BL group spent a significantly longer time in the open arms, indicating an alleviation of anxiety-like behaviors (**Figure 5A**). In contrast, no significant difference was observed between the two groups regarding the total number of arm entries (**Figure 5B**), indicating that general locomotor activity was unaffected by BL administration. Furthermore, evaluation of the frequency of head-dips, an indicator of exploratory behavior, revealed that the BL group exhibited a significantly higher frequency compared to the Control group (**Figure 5C**). Taken together, these indices strongly support the specific anxiolytic-like effect of BL.

### 3.4 Investigation of the mechanism underlying the ameliorative effects of BL on male menopausal symptoms

**Figure 6:**
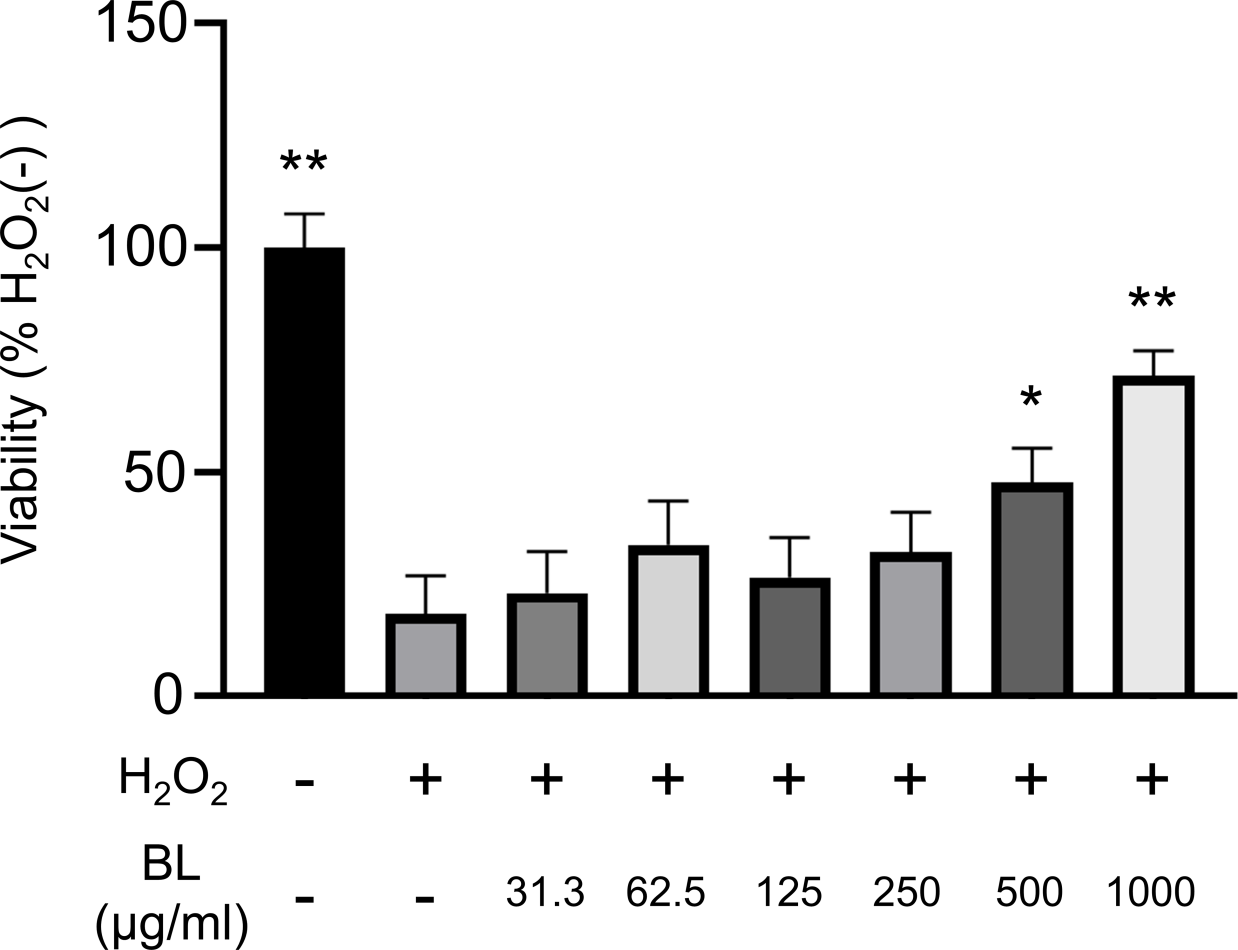
Protective effect of treatment of bee larvae extract against hydrogen peroxide-induced oxidative stress. NB1RGB cells were pre-treated with BL for 24 h. Subsequently, the cells were treated with 200 µM of hydrogen peroxide (H_2_O_2_) for 1 hour, washed with PBS, and cultured in fresh medium for an additional 24 h. Cell viability was measured using the CellTiter-Glo 2.0 assay. Data are presented as the mean ± standard error of the mean (SEM) (*n* = 5). Statistical analysis was performed using one-way analysis of variance (ANOVA) followed by Dunnett’s *post hoc* test for multiple comparisons against the H_2_O_2_ alone group. *P < 0.05, **P < 0.01.

The results from the animal studies suggested that BL supplementation exerts ameliorative effects on erectile function and anxiety-like symptoms in male menopausal models. Consequently, the cytoprotective effects of BL against oxidative stress, a known contributing factor to the etiology of male menopausal symptoms, were investigated. Exposure of NB1RGB cells to oxidative stress induced by 200 µM hydrogen peroxide H_2_O_2_ resulted in significant cytotoxicity; however, the addition of BL extract inhibited cell death in a dose-dependent manner (**Figure 6**). Compared to the H_2_O_2_**-**treated control group, the BL extract significantly preserved cell viability at concentrations of 500 (*P* < 0.05) and 1000 µg/mL (*P* < 0.01).

## 4 Discussion

The present study investigated the efficacy of BL on sexual function and psychological symptoms using animal models that mimic the pathology of male menopause. The investigation using an aged rat model suggested that BL supplementation can attenuate the age-associated decline in ejaculation frequency without affecting androgen levels. Based on these findings, the primary objective of this study was to elucidate the mechanism of action of BL using a castrated mouse model, an established model of male menopause. We intentionally utilized distinct models across two rodent species to ensure the robustness of our findings. While the aged rat model closely reflects the natural, gradual decline in sexual function, the castrated mouse model provides a highly standardized approach for assessing acute androgen deficiency and anxiety-like behaviors. Demonstrating the efficacy of BL across both models highlights its broad physiological applicability. Given that the beneficial effects of BL on sexual function were observed after a 4-week administration period in the preceding aged rat study, a corresponding 4-week post-castration period was selected as the primary evaluation window for the mouse experiments. It is well documented in rodents that testosterone levels decline rapidly following castration, leading to a time-dependent decline in sexual behavior, including decreased frequencies of mounts, intromissions, and ejaculations, as well as increased latency to intromission (Clemens et al., 1988; Smith et al., 1977; Wee et al., 1988; Hull and Dominguez, 2007). In the present study, BL administration over a 12-week period showed no effects on body weight, food intake, or sexual behavior in intact (pre-castration) mice. These results suggest that BL does not disrupt physiological homeostasis in healthy individuals but exerts its modulatory effects specifically under conditions where physiological balance is compromised, such as in male menopausal symptoms.

In the present study utilizing the castrated mouse model, a significant shortening of intromission latency, a reliable index of erectile function, was observed in the BL group compared to the Control group. This finding implies a facilitation of the initiation of erection. Conversely, no significant effects were observed on mount frequency, an indicator of sexual motivation, or on intromission frequency, which reflects the maintenance of erectile function. Collectively, these results suggest that BL does not modulate sexual desire (libido) *per se* but rather acts specifically on the physiological onset of erectile function.

This specific effect stands in contrast to the results obtained from the preceding aged rat model. In the rat model, BL administration suppressed the age-associated decline in ejaculation frequency but did not result in significant shortening of indicators such as intromission latency. This discrepancy is likely attributable to differences in the pathological profiles of the two models; whereas the primary vulnerability in the aged rat model lies in the decline of the endurance and recovery of sexual behavior, the castrated mouse model revealed the initiation of erection as the most sensitive indicator. The effect on erectile function in the castrated mouse model suggests a temporal aspect to the efficacy of BL. Significant shortening of intromission latency was observed at 2 weeks post-castration (week 14); however, this effect was not sustained at 4 weeks post-castration (week 16), when no significant difference was observed between groups. This suggests that the action of BL may not be a chronic or maintenance effect, but rather that it acts particularly against the acute physiological stress induced by androgen deficiency, potentially delaying the progression of the pathology. The erection-specific nature of the observed effects supports the hypothesis that the mechanism of action may involve non-hormonal pathways. Clinical trials evaluating the impact of BL administration on sexual function have similarly reported improvements in erectile function in middle-aged and elderly men, while revealing that circulating testosterone levels remained unaffected (Keishi et al., 2020). Generally, a decline in testosterone correlates with diminished sexual motivation, leading to a decrease in mount frequency (Clemens et al., 1988). However, in the present male menopause model, no change was observed in mount frequency—an index of sexual motivation. This raises the possibility that BL might act independently of direct androgen receptor activation, and instead directly targets downstream physiological processes, particularly vascular function. It is well established that the rapid decline in androgens due to castration exerts deleterious effects on both sexual function and mental state. Specifically, androgen depletion alters dopamine metabolism in the reward system of the central nervous system (including the nucleus accumbens and ventral tegmental area), which is involved in sexual motivation and pleasure, thereby suppressing behaviors such as mounting and ejaculation (Smith et al., 1977). Concurrently, it induces a state of systemic chronic inflammation and vascular endothelial dysfunction, leading to impaired vasodilatory function of the corpus cavernosum and potentially cerebrovascular disorders (Babcock et al., 2022; Kelly and Jones, 2013). These alterations contribute to the pathogenesis of both physical (erectile) and psychological (anxiety, depression) symptoms. In the present study, the BL group exhibited a suppression of the prolongation of intromission latency—an indicator of the initiation of erectile function—while simultaneously showing a significant alleviation of castration-induced anxiety-like behaviors in the elevated plus maze test. These results suggest that BL may target a common pathology within the vascular and neural systems induced by androgen deficiency, thereby concurrently improving both erectile function and psychiatric symptoms.

Erectile dysfunction (ED) and anxiety are hallmark symptoms of male menopause, and both are intricately linked to oxidative stress (Ferrini et al., 2017; Kaltsas et al., 2024). Erectile function is critically dependent on vascular endothelial integrity, and it is well established that aging, lifestyle-related diseases, and androgen decline exacerbate oxidative stress within vascular tissues (Ferrini et al., 2017; Agarwal et al., 2006; Kaltsas et al., 2024). Excessive oxidative stress impairs the production and bioavailability of nitric oxide (NO), a vasodilator essential for erection, thereby acting as a primary driver of ED pathophysiology. Similarly, anxiety and psychological stress are known to elevate oxidative stress levels within the brain. Cerebral neurons are particularly vulnerable to oxidative stress due to their high oxygen consumption and lipid-rich composition (Bouayed et al., 2009). Oxidative damage to neurons disrupts the homeostasis of neurotransmitter systems, such as the GABAergic and serotonergic pathways. This disruption is postulated to manifest as psychiatric symptoms including anxiety and depression (Bouayed et al., 2009). In models of male menopause, the coexistence of physical and psychological symptoms associated with androgen deficiency suggests a common pathological basis driven by elevated oxidative stress.

*In vitro* assays demonstrated that BL exerts potent protective effects against oxidative stress-induced cytotoxicity triggered by agents such as hydrogen peroxide. The potent cytoprotective activity of BL powder may be attributed to its rich nutritional profile. BL is known to contain an abundance of antioxidant components, including polyphenols and flavonoids, as well as trace elements such as zinc and selenium, which serve as essential cofactors for endogenous antioxidant enzymes (Guiné et al., 2025; Abd El-Wahed et al., 2024; Inci et al., 2023). Therefore, these constituents may have acted synergistically to enhance cellular defense mechanisms. The findings from the *in vitro* assays provide a plausible mechanistic explanation for the improvements in erectile function and anxiety-like behaviors observed *in vivo*. Specifically, this suggests that the antioxidant properties of BL directly suppressed oxidative stress induced by androgen deficiency within both vascular tissues and the nervous system. In vascular tissues, the mitigation of oxidative stress likely restored vascular endothelial function, thereby improving blood flow to the corpus cavernosum and enhancing erectile function. In parallel, within the nervous system, the suppression of oxidative stress may have contributed to neuroprotection and the maintenance of glial (astrocyte and microglia) homeostasis. This, in turn, would restore the balance of neurotransmitters, leading to the alleviation of anxiety symptoms. Clinical research further supports the notion that antioxidant intake alleviates anxiety symptoms. A meta-analysis integrating multiple randomized controlled trials demonstrated that antioxidant supplementation significantly improves anxiety states (Wang et al., 2022); this is consistent with the results of the present study, where the antioxidant action of BL contributed to the reduction of anxiety-like behaviors. Consequently, these findings suggest that BL exerts dual beneficial effects on both physical and psychological aspects of andropause-like symptoms *via* antioxidant-mediated vascular and neuroprotective mechanisms.

The present study has several limitations that should be addressed in future research. First, while our *in vitro* assays confirmed that BL exhibits potent protective effects against hydrogen peroxide-induced oxidative stress, biochemical verification was not performed to determine whether this translates to a reduction in oxidative stress markers within the target tissues—specifically, the corpus cavernosum and brain regions associated with anxiety—*in vivo*. Consequently, the proposition that BL directly mitigates oxidative stress in these tissues remains a hypothesis requiring further biochemical validation. Second, the specific active constituents within the enzyme-treated BL powder responsible for the observed antioxidant effects and behavioral improvements have not yet been determined. Identifying these bioactive principles represents a critical challenge for elucidating the molecular mechanism of action and for advancing future clinical applications. A third limitation concerns the potential influence on androgen levels in the mouse model. Although the mechanistic hypothesis is built upon the premise of hormone independence—derived from human clinical studies and the aged rat data—plasma testosterone levels were not directly measured in the castrated mice used in this specific experiment. Therefore, the possibility of an effect on trace residual androgen levels cannot entirely excluded, and this remains a subject for future investigation. Finally, castration is an established method for studying androgen-dependent sexual behavior; however, it represents a phenotype of acute and severe androgen deficiency, which differs from the gradual, age-associated decline observed in humans. Therefore, to extrapolate these findings to chronic male menopausal symptoms in humans, further validation using aging models is warranted.

## 5 Conclusion

In conclusion, the present study demonstrates that BL ameliorates andropause-like symptoms, specifically erectile dysfunction and anxiety-like behaviors, in male menopausal rodent models. Our findings suggest that this efficacy may involve non-hormonal pathways, potentially through vascular and neuroprotective effects resulting from the potent antioxidant activity of BL. These results highlight the potential of BL as a functional food for managing andropause-like symptoms associated with male reproductive aging.

## Data Availability

The original contributions presented in the study are included in the article/Supplementary Material. Further inquiries can be directed to the corresponding author.
